# Supplementary material for: Hospital delivery and neonatal mortality in 37 countries in sub-Saharan Africa and South Asia: An ecological study
Source: PLoS Med. 2021 Dec 1;18(12):e1003843. doi: 10.1371/journal.pmed.1003843 (PMC8635398; doi:10.1371/journal.pmed.1003843)
Supplement: S1 Table — (DOCX) [file pmed.1003843.s002.docx]

**S1 Table**. C-section rates by facility level and country

| Country | Percent C-section among hospital births | N hospital births | Percent C-sections among clinic births | N clinic births | Percent C-section among uncategorized facility births | N uncategorized births |
| --- | --- | --- | --- | --- | --- | --- |
| Afghanistan | 6% | 11958 | 2% | 2641 | 6% | 387 |
| Angola | 10% | 4779 | 1% | 1231 | 0% | 266 |
| Bangladesh | 64% | 1816 | 3% | 24 | 2% | 15 |
| Benin | 12% | 4009 | 3% | 7072 | 1% | 249 |
| Burkina Faso | 14% | 739 | 2% | 9522 | 2% | 47 |
| Burundi | 23% | 3221 | 0% | 7821 | 1% | 586 |
| Cameroon | 8% | 4082 | 4% | 3198 | 2% | 102 |
| Chad | 9% | 1736 | 5% | 1813 | 0% | 45 |
| Congo, Dem. Rep. | 14% | 4428 | 3% | 8453 | 2% | 1181 |
| Congo, Rep. | 8% | 4955 | 2% | 2777 | 0% | 53 |
| Côte d'Ivoire | 8% | 1564 | 3% | 2613 | 0% | 99 |
| Ethiopia | 22% | 1350 | 1% | 1933 | 3% | 203 |
| Gabon | 11% | 4158 | 9% | 615 | 7% | 241 |
| Ghana | 22% | 2700 | 6% | 1362 | 0% | 25 |
| Guinea | 12% | 987 | 3% | 1750 | 0% | 23 |
| India | 30% | 115459 | 7% | 79907 | 0% | 631 |
| Kenya | 19% | 7357 | 3% | 3012 | 2% | 221 |
| Lesotho | 15% | 1829 | 2% | 361 | 21% | 166 |
| Liberia | 10% | 2125 | 2% | 1822 | 0% | 70 |
| Madagascar | 13% | 1087 | 2% | 3233 | 0% | 197 |
| Malawi | 15% | 6604 | 2% | 9020 | 6% | 549 |
| Mali | 11% | 597 | 3% | 5706 | 2% | 70 |
| Mozambique | 11% | 2597 | 4% | 3952 | 9% | 315 |
| Namibia | 17% | 4017 | 4% | 283 | 8% | 32 |
| Nepal | 22% | 1930 | 2% | 787 | 4% | 244 |
| Niger | 18% | 1100 | 1% | 3260 | 0% | 136 |
| Nigeria | 7% | 8390 | 2% | 3041 | 0% | 122 |
| Pakistan | 35% | 6571 | 2% | 47 | 8% | 86 |
| Rwanda | 46% | 2185 | 1% | 4962 | 0% | 117 |
| Senegal | 22% | 1345 | 2% | 7567 | 0% | 226 |
| Sierra Leone | 13% | 1721 | 5% | 4939 | 2% | 50 |
| Swaziland | 12% | 1831 | 3% | 238 | 4% | 21 |
| Tanzania | 17% | 3327 | 1% | 3001 | 0% | 149 |
| Togo | 19% | 1728 | 2% | 3004 | 2% | 52 |
| Uganda | 15% | 5044 | 2% | 6118 | 0% | 262 |
| Zambia | 16% | 3011 | 2% | 5549 | 7% | 748 |
| Zimbabwe | 13% | 2662 | 0% | 1805 | 6% | 594 |
