# Supplementary material for: Hospital delivery and neonatal mortality in 37 countries in sub-Saharan Africa and South Asia: An ecological study
Source: PLoS Med. 2021 Dec 1;18(12):e1003843. doi: 10.1371/journal.pmed.1003843 (PMC8635398; doi:10.1371/journal.pmed.1003843)
Supplement: S2 Table — (DOCX) [file pmed.1003843.s003.docx]

**S2 Table.** Percent of births missing key variables by country

|  | Deaths | Birth location | Small at birth | Antenatal care visits | Multiple | Maternal Age | Urban | First birth | Birth interval | Education |
| --- | --- | --- | --- | --- | --- | --- | --- | --- | --- | --- |
| Afghanistan | 0% | 0% | 3% | 1% | 0% | 0% | 0% | 0% | 0% | 0% |
| Angola | 0% | 0% | 5% | 1% | 0% | 0% | 0% | 0% | 0% | 0% |
| Bangladesh | 0% | 0% | 0% | 0% | 0% | 0% | 0% | 0% | 0% | 0% |
| Benin | 0% | 2% | 2% | 2% | 0% | 0% | 0% | 0% | 0% | 0% |
| Burkina Faso | 0% | 0% | 1% | 0% | 0% | 0% | 0% | 0% | 0% | 0% |
| Burundi | 0% | 0% | 0% | 0% | 0% | 0% | 0% | 0% | 0% | 0% |
| Cameroon | 0% | 0% | 1% | 1% | 0% | 0% | 0% | 0% | 0% | 0% |
| Chad | 0% | 0% | 1% | 1% | 0% | 0% | 0% | 0% | 0% | 0% |
| Congo, Dem. Rep. | 0% | 0% | 2% | 0% | 0% | 0% | 0% | 0% | 0% | 0% |
| Congo, Rep. | 0% | 3% | 1% | 0% | 0% | 0% | 0% | 0% | 0% | 0% |
| Côte d'Ivoire | 0% | 0% | 3% | 1% | 0% | 0% | 0% | 0% | 0% | 0% |
| Ethiopia | 0% | 0% | 1% | 0% | 0% | 0% | 0% | 0% | 0% | 0% |
| Gabon | 0% | 2% | 4% | 1% | 0% | 0% | 0% | 0% | 0% | 0% |
| Ghana | 0% | 0% | 0% | 0% | 0% | 0% | 0% | 0% | 0% | 0% |
| Guinea | 0% | 0% | 0% | 0% | 0% | 0% | 0% | 0% | 0% | 0% |
| India | 0% | 0% | 2% | 1% | 0% | 0% | 0% | 0% | 0% | 0% |
| Kenya | 0% | 6% | 53% | 0% | 0% | 0% | 0% | 0% | 0% | 0% |
| Lesotho | 0% | 0% | 0% | 1% | 0% | 0% | 0% | 0% | 0% | 0% |
| Liberia | 0% | 0% | 0% | 3% | 0% | 0% | 0% | 0% | 0% | 0% |
| Madagascar | 0% | 1% | 3% | 1% | 0% | 0% | 0% | 0% | 0% | 0% |
| Malawi | 0% | 0% | 1% | 0% | 0% | 0% | 0% | 0% | 0% | 0% |
| Mali | 0% | 0% | 5% | 2% | 0% | 0% | 0% | 0% | 0% | 0% |
| Mozambique | 0% | 1% | 4% | 1% | 0% | 0% | 0% | 0% | 0% | 0% |
| Namibia | 0% | 0% | 1% | 19% | 0% | 0% | 0% | 0% | 0% | 0% |
| Nepal | 0% | 0% | 0% | 0% | 0% | 0% | 0% | 0% | 0% | 0% |
| Niger | 0% | 0% | 3% | 0% | 0% | 0% | 0% | 0% | 0% | 0% |
| Nigeria | 0% | 1% | 2% | 2% | 0% | 0% | 0% | 0% | 0% | 0% |
| Pakistan | 0% | 0% | 0% | 0% | 0% | 0% | 0% | 0% | 0% | 0% |
| Rwanda | 0% | 0% | 0% | 0% | 0% | 0% | 0% | 0% | 0% | 0% |
| Senegal | 0% | 0% | 0% | 1% | 0% | 0% | 0% | 0% | 0% | 0% |
| Sierra Leone | 0% | 1% | 3% | 9% | 0% | 0% | 0% | 0% | 0% | 0% |
| Swaziland | 0% | 0% | 3% | 2% | 0% | 0% | 0% | 0% | 0% | 0% |
| Tanzania | 0% | 0% | 1% | 0% | 0% | 0% | 0% | 0% | 0% | 0% |
| Togo | 0% | 0% | 1% | 0% | 0% | 0% | 0% | 0% | 0% | 0% |
| Uganda | 0% | 0% | 2% | 0% | 0% | 0% | 0% | 0% | 0% | 0% |
| Zambia | 0% | 0% | 1% | 1% | 0% | 0% | 0% | 0% | 0% | 0% |
| Zimbabwe | 0% | 0% | 0% | 0% | 0% | 0% | 0% | 0% | 0% | 0% |
