# Supplementary material for: Hospital delivery and neonatal mortality in 37 countries in sub-Saharan Africa and South Asia: An ecological study
Source: PLoS Med. 2021 Dec 1;18(12):e1003843. doi: 10.1371/journal.pmed.1003843 (PMC8635398; doi:10.1371/journal.pmed.1003843)
Supplement: S3 Table — (DOCX) [file pmed.1003843.s004.docx]

**S3 Table.** Bonferroni adjusted models

P-values from the adjusted random intercept models presented in Tables 3 and 4 are multiplied by three to account for the three different outcomes of early neonatal mortality, neonatal mortality and post-natal mortality. Only the primary exposure is shown.

Table 3 with Bonferroni adjusted p values

|  | Early neonatal death (per 1000 births) | | Neonatal death (per 1000 births) | | Post-neonatal death (per 1000 births) | |
| --- | --- | --- | --- | --- | --- | --- |
|  | Coef. | p value | Coef. | p value | Coef. | p value |
| Hospital % among facility deliveries | -12 | 0.00 | -14.9 | 0.00 | -4.4 | 0.12 |

Table 4 with Bonferroni adjusted p values

|  | Early neonatal death (per 1000 births) | | Neonatal death (per 1000 births) | | Post-neonatal death (per 1000 births) | |
| --- | --- | --- | --- | --- | --- | --- |
|  | Coef. | p value | Coef. | p value | Coef. | p value |
| All facility % | 8.9 | 0.06 | 10.3 | 0.06 | -4.9 | 0.75 |
