# Supplementary material for: Hospital delivery and neonatal mortality in 37 countries in sub-Saharan Africa and South Asia: An ecological study
Source: PLoS Med. 2021 Dec 1;18(12):e1003843. doi: 10.1371/journal.pmed.1003843 (PMC8635398; doi:10.1371/journal.pmed.1003843)
Supplement: S4 Table — (DOCX) [file pmed.1003843.s005.docx]

**S4 Table**. Interaction models specification and full model results

$$NNM_{ij}=\beta_{0i}+\beta_{1}\%Hosp_{ij}+ \beta_{2}{CV}_{i}+\beta_{3}\%Hosp_{ij}*{CV}_{i}+\beta_{4}\gamma_{ij}+\epsilon_{ij}$$

$$\beta_{0i}= \beta_{0}+b_{i}$$

$$where b_{i}\sim N\left( 0,\sigma_{b}^{2} \right) and \epsilon_{ij}\sim N(0,\sigma_{\epsilon}^{2})$$

Where $NNM_{ij}$ is neonatal mortality in sub-national region *j* in country *I*; *%Hosp* is the share of facility deliveries occurring in a hospital; *CV* is one of the binary contextual variables (middle income country, country in South Asia or Urban region) and $\gamma_{ij}$ is the full set of covariates.

Hospital delivery associations with early neonatal mortality per 1000 births

|  | Country income interaction | | | Global region interaction | | | Urban regions interaction | | |
| --- | --- | --- | --- | --- | --- | --- | --- | --- | --- |
|  | Coef. | p value | 95% CI | Coef. | p value | 95% CI | Coef. | p value | 95% CI |
| Hospital % among facility deliveries | 1.0 | 0.8 | [-7.2,9.2] | 3.5 | 0.38 | [-4.3,11.3] | -11.6 | 0 | [-15.3,-7.9] |
| Middle income country (vs. low income) | 13.5 | 0 | [6.8,20.2] | 1.8 | 0.39 | [-2.3,5.9] | 6.4 | 0 | [2.0,10.8] |
| Middle income, Hospital % interaction | -15.7 | 0 | [-24.8,-6.6] |  |  |  |  |  |  |
| South Asia (vs. Sub-Saharan Africa) | 3.9 | 0.34 | [-4.1,11.9] | 19.1 | 0 | [10.7,27.4] | 5.9 | 0.1 | [-1.1,13.0] |
| South Asia, Hospital % interaction |  |  |  | -21.5 | 0 | [-27.4,-15.7] |  |  |  |
| Urban regions (vs. rural) |  |  |  |  |  |  | 3.9 | 0.21 | [-2.1,9.9] |
| Urban, Hospital % interaction |  |  |  |  |  |  | -5.8 | 0.11 | [-12.8,1.3] |
| All facility % | 5.9 | 0.06 | [-0.2,11.9] | 4.4 | 0.21 | [-2.5,11.4] | 6.4 | 0.03 | [0.5,12.4] |
| Small at birth % | 6.7 | 0.06 | [-0.4,13.9] | 5.8 | 0.1 | [-1.2,12.8] | 6.9 | 0.06 | [-0.3,14.2] |
| Antenatal care visit median | -0.3 | 0.1 | [-0.6,0.1] | -0.1 | 0.39 | [-0.4,0.1] | -0.4 | 0.06 | [-0.8,0.0] |
| Urban % | -0.7 | 0.68 | [-3.9,2.5] | -1.3 | 0.4 | [-4.2,1.7] | 0.9 | 0.72 | [-3.9,5.6] |
| Multiple birth % | 15.3 | 0 | [11.5,19.1] | 16.1 | 0 | [11.9,20.3] | 16.2 | 0 | [11.9,20.5] |
| Average maternal age | -0.1 | 0.8 | [-0.6,0.5] | -0.1 | 0.77 | [-0.7,0.5] | -0.1 | 0.85 | [-0.6,0.5] |
| First birth % | -7 | 0.27 | [-19.6,5.5] | -7.1 | 0.25 | [-19.2,5.0] | -8.9 | 0.15 | [-20.9,3.2] |
| Less than 2 year birth interval % | 22 | 0.02 | [3.6,40.4] | 22.7 | 0.01 | [4.7,40.7] | 21.4 | 0.02 | [3.3,39.5] |
| Mother's primary education % | 2.1 | 0.53 | [-4.5,8.6] | 3.7 | 0.25 | [-2.6,10.0] | 2.1 | 0.53 | [-4.5,8.7] |
| Mother's secondary education or higher % | -13.9 | 0 | [-21.3,-6.6] | -14.1 | 0 | [-21.7,-6.6] | -13.7 | 0 | [-21.1,-6.3] |
| Average annual income | 0 | 0.96 | [-1.8,1.9] | 0.6 | 0.56 | [-1.5,2.7] | -0.2 | 0.81 | [-2.2,1.7] |

Facility delivery associations with early neonatal mortality per 1000 births

|  | Country income interaction | | | Global region interaction | | | Urban regions interaction | | |
| --- | --- | --- | --- | --- | --- | --- | --- | --- | --- |
|  | Coef. | p value | 95% CI | Coef. | p value | 95% CI | Coef. | p value | 95% CI |
| All facility % | 13.0 | 0.0 | [1.7,24.2] | 16.0 | 0.0 | [5.4,26.6] | 9.5 | 0.01 | [2.2,16.9] |
| Middle income country (vs. low income) | 8.9 | 0.02 | [1.6,16.2] | 5.3 | 0.05 | [0.0,10.6] | 4.8 | 0.07 | [-0.3,9.9] |
| Middle income, Hospital % interaction | -6.5 | 0.17 | [-15.7,2.7] |  |  |  |  |  |  |
| South Asia (vs. Sub-Saharan Africa) | 4 | 0.32 | [-3.9,11.9] | 11.4 | 0.01 | [3.1,19.7] | 4.5 | 0.28 | [-3.6,12.6] |
| South Asia, Hospital % interaction |  |  |  | -15 | 0 | [-22.2,-7.7] |  |  |  |
| Urban regions (vs. rural) |  |  |  |  |  |  | 3.8 | 0.53 | [-8.0,15.5] |
| Urban, Hospital % interaction |  |  |  |  |  |  | -4.2 | 0.51 | [-17.0,8.5] |
| Small at birth % | 7.5 | 0.04 | [0.2,14.7] | 8.8 | 0.02 | [1.6,15.9] | 6.6 | 0.07 | [-0.6,13.9] |
| Antenatal care visit median | -0.5 | 0 | [-0.9,-0.2] | -0.4 | 0.01 | [-0.7,-0.1] | -0.6 | 0 | [-1.0,-0.2] |
| Urban % | -2 | 0.27 | [-5.4,1.5] | -2.8 | 0.05 | [-5.7,0.0] | -1.7 | 0.55 | [-7.3,3.9] |
| Multiple birth % | 16.8 | 0 | [12.6,21.1] | 16.1 | 0 | [12.3,20.0] | 16.7 | 0 | [12.5,20.9] |
| Average maternal age | -0.3 | 0.36 | [-0.9,0.3] | -0.4 | 0.26 | [-1.0,0.3] | -0.2 | 0.53 | [-0.8,0.4] |
| First birth % | -12.1 | 0.06 | [-24.4,0.3] | -5.8 | 0.46 | [-21.1,9.6] | -13.9 | 0.03 | [-26.1,-1.7] |
| Less than 2 year birth interval % | 16.9 | 0.04 | [1.1,32.7] | 20.4 | 0 | [7.3,33.5] | 16 | 0.06 | [-0.6,32.7] |
| Mother's primary education % | 0 | 0.99 | [-7.3,7.4] | -2.4 | 0.5 | [-9.5,4.6] | 1.4 | 0.68 | [-5.3,8.2] |
| Mother's secondary education or higher % | -15.3 | 0 | [-23.7,-7.0] | -17.7 | 0 | [-24.2,-11.2] | -15.2 | 0 | [-22.9,-7.6] |
| Average annual income | -1.8 | 0.27 | [-4.9,1.4] | -1.5 | 0.37 | [-4.7,1.7] | -2 | 0.21 | [-5.2,1.2] |

Models also include survey year fixed effects for years 2009-2018. Small at birth is birth weight <2500 grams or mother’s report at birth. Mother’s primary education is completed only primary education; mother’s secondary education or higher is completed secondary or higher education. Average annual income based on estimates from the International Center for Equity in Health, they are in log 2011 international dollars adjusted at purchasing power parity
