# Supplementary material for: Hospital delivery and neonatal mortality in 37 countries in sub-Saharan Africa and South Asia: An ecological study
Source: PLoS Med. 2021 Dec 1;18(12):e1003843. doi: 10.1371/journal.pmed.1003843 (PMC8635398; doi:10.1371/journal.pmed.1003843)
Supplement: S5 Table — (DOCX) [file pmed.1003843.s006.docx]

**S5 Table.** Within-country variation in place of delivery

|  | Share of hospital delivery among facility deliveries | | Facility delivery among all births | |
| --- | --- | --- | --- | --- |
| Country | Min region | Max region | Min region | Max region |
| Afghanistan | 29% | 98% | 1% | 84% |
| Angola | 54% | 96% | 17% | 88% |
| Bangladesh | 96% | 99% | 23% | 55% |
| Benin | 18% | 69% | 62% | 100% |
| Burkina Faso | 0% | 25% | 36% | 97% |
| Burundi | 5% | 72% | 81% | 96% |
| Cameroon | 33% | 79% | 23% | 99% |
| Chad | 19% | 66% | 4% | 69% |
| Congo, Dem. Rep. | 19% | 65% | 61% | 98% |
| Congo, Rep. | 39% | 95% | 72% | 100% |
| Côte d'Ivoire | 20% | 54% | 27% | 91% |
| Ethiopia | 16% | 73% | 15% | 97% |
| Gabon | 63% | 95% | 61% | 98% |
| Ghana | 32% | 86% | 36% | 93% |
| Guinea | 11% | 67% | 19% | 81% |
| India | 6% | 100% | 10% | 100% |
| Kenya | 31% | 92% | 20% | 94% |
| Lesotho | 63% | 88% | 61% | 84% |
| Liberia | 42% | 80% | 48% | 65% |
| Madagascar | 5% | 42% | 9% | 69% |
| Malawi | 12% | 78% | 86% | 97% |
| Mali | 4% | 38% | 24% | 97% |
| Mozambique | 17% | 72% | 28% | 94% |
| Namibia | 86% | 99% | 73% | 99% |
| Nepal | 35% | 83% | 37% | 71% |
| Niger | 7% | 48% | 20% | 87% |
| Nigeria | 45% | 93% | 5% | 92% |
| Pakistan | 96% | 99% | 35% | 84% |
| Rwanda | 23% | 51% | 91% | 95% |
| Senegal | 5% | 35% | 49% | 96% |
| Sierra Leone | 15% | 63% | 38% | 74% |
| Swaziland | 73% | 95% | 66% | 80% |
| Tanzania | 22% | 89% | 42% | 95% |
| Togo | 25% | 60% | 52% | 97% |
| Uganda | 23% | 74% | 57% | 95% |
| Zambia | 14% | 35% | 48% | 90% |
| Zimbabwe | 32% | 71% | 69% | 95% |
