# Supplementary material for: Hospital delivery and neonatal mortality in 37 countries in sub-Saharan Africa and South Asia: An ecological study
Source: PLoS Med. 2021 Dec 1;18(12):e1003843. doi: 10.1371/journal.pmed.1003843 (PMC8635398; doi:10.1371/journal.pmed.1003843)
Supplement: S6 Table — (DOCX) [file pmed.1003843.s007.docx]

**S6 Table.** Unadjusted main model results

|  |  | | | | |
| --- | --- | --- | --- | --- | --- |
|  | Share of facility deliveries in hospitals | | Share of deliveries in any facility | |  |
|  | Coef. | 95% CI | Coef. | 95% CI |  |
| Main model |  |  |  |  |  |
| Early neonatal death per 1000 births | -17.8 | [-30.3,-5.2] | -10.2 | [-21.0,0.6] |  |
| Alternate outcomes |  |  |  |  |  |
| Neonatal death per 1000 births | -21 | [-35.4,-6.7] | -12.7 | [-23.6,-1.8] |  |
| Post-neonatal death per 1000 births | -10.2 | [-12.1,-8.2] | -14.3 | [-18.3,-10.4] |  |
