# Supplementary material for: Hospital delivery and neonatal mortality in 37 countries in sub-Saharan Africa and South Asia: An ecological study
Source: PLoS Med. 2021 Dec 1;18(12):e1003843. doi: 10.1371/journal.pmed.1003843 (PMC8635398; doi:10.1371/journal.pmed.1003843)
Supplement: S7 Table — (DOCX) [file pmed.1003843.s008.docx]

**S7 Table**. Association between percent of all deliveries in hospital and neonatal mortality

|  | Early neonatal death (per 1000 births) | | | Neonatal death (per 1000 births) | | | Post-neonatal death (per 1000 births) | | |
| --- | --- | --- | --- | --- | --- | --- | --- | --- | --- |
|  | Coef. | p value | 95% CI | Coef. | p value | 95% CI | Coef. | p value | 95% CI |
| Hospital % among facility deliveries | -15.8 | 0.00 | -19.3,-12.3 | -19.5 | 0.00 | -23.6,-15.4 | -4 | 0.10 | -8.7,0.7 |
| All facility % | 14.9 | 0.00 | 8.4,21.5 | 17.6 | 0.00 | 9.3,25.9 | -3.5 | 0.38 | -11.2,4.3 |
| Small at birth % | 6.7 | 0.05 | -0.1,13.6 | 10.7 | 0.00 | 3.3,18.0 | 2.6 | 0.64 | -8.2,13.3 |
| Antenatal care visit median | -0.3 | 0.14 | -0.7,0.1 | -0.4 | 0.06 | -0.9,0.0 | -0.2 | 0.27 | -0.6,0.2 |
| Multiple birth % | 16.2 | 0.00 | 12.2,20.2 | 18.5 | 0.00 | 15.6,21.5 | 4.4 | 0.04 | 0.1,8.7 |
| Average maternal age | -0.1 | 0.74 | -0.6,0.5 | -0.1 | 0.85 | -0.7,0.5 | 0.1 | 0.67 | -0.4,0.6 |
| Urban % | 0 | 0.99 | -3.2,3.2 | 0 | 0.98 | -3.2,3.1 | 1.7 | 0.14 | -0.6,4.0 |
| First birth % | -7.3 | 0.26 | -20.0,5.4 | -7.9 | 0.21 | -20.3,4.6 | -8.4 | 0.37 | -27.0,10.1 |
| Less than 2 year birth interval % | 22.4 | 0.01 | 5.1,39.7 | 28 | 0.01 | 7.4,48.7 | 20 | 0.01 | 5.0,35.0 |
| Mother's primary education % | 1.3 | 0.68 | -5.1,7.8 | -0.3 | 0.93 | -7.1,6.4 | 3.1 | 0.42 | -4.3,10.5 |
| Mother's secondary education or higher % | -13.5 | 0.00 | -21.9,-5.2 | -16.2 | 0 | -25.5,-6.8 | -1.5 | 0.29 | -4.2,1.3 |
| Average annual income | -0.1 | 0.93 | -2.2,2.0 | 0.7 | 0.45 | -1.2,2.6 | -1.3 | 0.3 | -3.6,1.1 |
| South Asia (vs. Sub-Saharan Africa) | 4.7 | 0.19 | -2.4,11.8 | 6.4 | 0.12 | -1.7,14.4 | -0.6 | 0.83 | -6.2,4.9 |
| Middle income country (vs. low income) | 5.7 | 0.01 | 1.3,10.0 | 5.8 | 0.02 | 1.0,10.6 | -3.6 | 0.08 | -7.5,0.4 |
| N | 1143 |  |  | 1143 |  |  | 1143 |  |  |
