# Supplementary material for: Hospital delivery and neonatal mortality in 37 countries in sub-Saharan Africa and South Asia: An ecological study
Source: PLoS Med. 2021 Dec 1;18(12):e1003843. doi: 10.1371/journal.pmed.1003843 (PMC8635398; doi:10.1371/journal.pmed.1003843)
Supplement: S8 Table — (DOCX) [file pmed.1003843.s009.docx]

**S8 Table.** Robustness check including anemia as covariate in subset of study countries

|  | Early neonatal death (per 1000 births) | | | Neonatal death (per 1000 births) | | | Post-neonatal death (per 1000 births) | | |
| --- | --- | --- | --- | --- | --- | --- | --- | --- | --- |
|  | Coef. | p value | 95% CI | Coef. | p value | 95% CI | Coef. | p value | 95% CI |
| Hospital % among facility deliveries | -12.2 | 0.00 | [-16.4,-7.9] | -15.4 | 0.00 | [-21.4,-9.4] | -5.3 | 0.00 | [-8.6,-1.9] |
| All facility % | 5.6 | 0.02 | [0.9,10.3] | 5.8 | 0.05 | [-0.1,11.6] | -5.6 | 0.16 | [-13.4,2.2] |
| Small at birth % | 5.7 | 0.07 | [-0.4,11.9] | 10.2 | 0.00 | [4.9,15.5] | 6.9 | 0.11 | [-1.5,15.3] |
| Antenatal care visit median | -0.5 | 0.09 | [-1.1,0.1] | -0.7 | 0.03 | [-1.3,-0.1] | -0.1 | 0.53 | [-0.5,0.3] |
| Multiple birth % | 16 | 0.00 | [11.3,20.7] | 17.9 | 0.00 | [14.6,21.3] | 5 | 0.06 | [-0.3,10.3] |
| Average maternal age | -0.3 | 0.42 | [-1.1,0.5] | -0.3 | 0.52 | [-1.2,0.6] | 0.1 | 0.61 | [-0.3,0.5] |
| Urban % | 0.4 | 0.88 | [-4.5,5.2] | 0.2 | 0.92 | [-4.3,4.7] | 0.9 | 0.46 | [-1.5,3.3] |
| First birth % | -13.6 | 0.04 | [-26.7,-0.6] | -12.9 | 0.13 | [-29.5,3.8] | -12.1 | 0.19 | [-30.2,6.1] |
| Less than 2 year birth interval % | 12.8 | 0.33 | [-12.8,38.5] | 18.3 | 0.24 | [-12.2,48.9] | 14.4 | 0.02 | [2.6,26.3] |
| Anemia % | 10.9 | 0.01 | [3.2,18.6] | 12.1 | 0.00 | [4.9,19.4] | 4.6 | 0.36 | [-5.3,14.5] |
| Mother's primary education % | -1.5 | 0.75 | [-10.5,7.6] | -2.5 | 0.62 | [-12.6,7.5] | 0.2 | 0.95 | [-6.7,7.2] |
| Mother's secondary education or higher % | -15.5 | 0.00 | [-20.9,-10.1] | -17.6 | 0.00 | [-24.3,-11.0] | -1.7 | 0.24 | [-4.5,1.1] |
| Average annual income | 0.6 | 0.51 | [-1.1,2.2] | 1.5 | 0.05 | [0.0,3.0] | -0.6 | 0.56 | [-2.8,1.5] |
| South Asia (vs. Sub-Saharan Africa) | 3.6 | 0.24 | [-2.3,9.5] | 4.3 | 0.20 | [-2.3,11.0] | 1.8 | 0.64 | [-5.5,9.0] |
| Middle income country (vs. low income) | 6.9 | 0.01 | [1.5,12.2] | 7 | 0.02 | [1.1,13.0] | -3.7 | 0.18 | [-9.1,1.7] |
| N | 958 |  |  | 958 |  |  | 958 |  |  |
|  |  |  |  |  |  |  |  |  |  |
|  | Early neonatal death (per 1000 births) | | | Neonatal death (per 1000 births) | | | Post-neonatal death (per 1000 births) | | |
|  | Coef. | p value | 95% CI | Coef. | p value | 95% CI | Coef. | p value | 95% CI |
| All facility % | 8.0 | 0.00 | [3.0,13.0] | 10.8 | 0.00 | [5.0,16.5] | -4.5 | 0.26 | [-12.4,3.4] |
| Small at birth % | 7.6 | 0.02 | [1.1,14.0] | 12.7 | 0.00 | [7.6,17.9] | 8.2 | 0.04 | [0.2,16.1] |
| Antenatal care visit median | -0.8 | 0.00 | [-1.2,-0.3] | -1 | 0.00 | [-1.5,-0.6] | -0.2 | 0.19 | [-0.6,0.1] |
| Multiple birth % | 16.2 | 0.00 | [11.5,20.9] | 18.5 | 0.00 | [15.3,21.7] | 5 | 0.05 | [-0.1,10.0] |
| Average maternal age | -0.5 | 0.24 | [-1.2,0.3] | -0.3 | 0.41 | [-1.1,0.4] | 0.1 | 0.79 | [-0.4,0.5] |
| Urban % | -1.8 | 0.47 | [-6.9,3.2] | -2.3 | 0.36 | [-7.0,2.5] | 0.1 | 0.96 | [-2.5,2.6] |
| First birth % | -18 | 0.00 | [-29.9,-6.2] | -19 | 0.01 | [-32.6,-5.3] | -14.3 | 0.10 | [-31.3,2.7] |
| Less than 2 year birth interval % | 6.8 | 0.53 | [-14.6,28.3] | 13.2 | 0.29 | [-11.4,37.8] | 11.5 | 0.05 | [0.2,22.8] |
| Anemia % | 9.1 | 0.01 | [2.6,15.7] | 9.7 | 0.00 | [3.8,15.6] | 3.8 | 0.40 | [-5.1,12.8] |
| Mother's primary education % | -4.8 | 0.31 | [-13.9,4.3] | -6.4 | 0.28 | [-18.1,5.2] | -0.8 | 0.84 | [-8.5,6.9] |
| Mother's secondary education or higher % | -18.9 | 0.00 | [-23.5,-14.3] | -21.6 | 0.00 | [-26.1,-17.0] | -2.8 | 0.14 | [-6.5,0.9] |
| Average annual income | -1 | 0.56 | [-4.3,2.3] | -1 | 0.57 | [-4.6,2.5] | -1.4 | 0.11 | [-3.2,0.3] |
| South Asia (vs. Sub-Saharan Africa) | 5.3 | 0.11 | [-1.2,11.8] | 7.8 | 0.05 | [0.2,15.4] | 2.4 | 0.57 | [-5.8,10.5] |
| Middle income country (vs. low income) | 4.9 | 0.05 | [0.0,9.7] | 4.5 | 0.12 | [-1.2,10.2] | -4.7 | 0.10 | [-10.4,0.9] |
| N | 958 |  |  | 958 |  |  | 958 |  |  |
