# Supplementary material for: Hospital delivery and neonatal mortality in 37 countries in sub-Saharan Africa and South Asia: An ecological study
Source: PLoS Med. 2021 Dec 1;18(12):e1003843. doi: 10.1371/journal.pmed.1003843 (PMC8635398; doi:10.1371/journal.pmed.1003843)
Supplement: S9 Table — (DOCX) [file pmed.1003843.s010.docx]

**S9 Table.** Robustness check excluding Kenya from analysis

|  | Early neonatal death (per 1000 births) | | | Neonatal death (per 1000 births) | | | Post-neonatal death (per 1000 births) | | |
| --- | --- | --- | --- | --- | --- | --- | --- | --- | --- |
|  | Coef. | p value | 95% CI | Coef. | p value | 95% CI | Coef. | p value | 95% CI |
| Hospital % among facility deliveries | -12.4 | 0 | [-16.0,-8.7] | -15.2 | 0 | [-20.3,-10.2] | -3.9 | 0.06 | [-7.9,0.1] |
| All facility % | 4.9 | 0.13 | [-1.5,11.2] | 5.2 | 0.18 | [-2.4,12.9] | -7 | 0.13 | [-16.0,2.1] |
| Small at birth % | 6.2 | 0.12 | [-1.6,14.0] | 10.1 | 0.02 | [1.8,18.4] | 3.5 | 0.56 | [-8.2,15.1] |
| Antenatal care visit median | -0.3 | 0.09 | [-0.7,0.1] | -0.5 | 0.03 | [-0.9,-0.1] | -0.1 | 0.51 | [-0.5,0.3] |
| Multiple birth % | 16.6 | 0 | [11.7,21.6] | 19.2 | 0 | [15.4,23.1] | 4.4 | 0.07 | [-0.3,9.0] |
| Average maternal age | -0.1 | 0.68 | [-0.7,0.5] | -0.1 | 0.75 | [-0.8,0.6] | 0.2 | 0.41 | [-0.2,0.6] |
| Urban % | -0.3 | 0.84 | [-3.6,3.0] | -0.3 | 0.84 | [-3.6,2.9] | 1.7 | 0.14 | [-0.5,4.0] |
| First birth % | -7.5 | 0.25 | [-20.1,5.1] | -8.1 | 0.22 | [-21.1,4.9] | -7.4 | 0.43 | [-25.8,11.1] |
| Less than 2 year birth interval % | 23.1 | 0.01 | [4.9,41.2] | 28.3 | 0.01 | [5.9,50.6] | 21.8 | 0.01 | [5.5,38.1] |
| Mother's primary education % | 2.9 | 0.46 | [-4.8,10.7] | 1.5 | 0.73 | [-6.9,9.9] | 0.8 | 0.85 | [-7.6,9.3] |
| Mother's secondary education or higher % | -13.9 | 0 | [-21.6,-6.2] | -16.7 | 0 | [-25.1,-8.3] | -1.9 | 0.17 | [-4.6,0.8] |
| Average annual income | 0.3 | 0.79 | [-1.8,2.3] | 1.1 | 0.23 | [-0.7,2.9] | -1.4 | 0.34 | [-4.3,1.5] |
| South Asia (vs. Sub-Saharan Africa) | 4.9 | 0.14 | [-1.6,11.4] | 6.7 | 0.08 | [-0.7,14.1] | -1.3 | 0.64 | [-6.5,4.0] |
| Middle income country (vs. low income) | 6.9 | 0 | [2.4,11.5] | 7.3 | 0 | [2.4,12.2] | -2.6 | 0.19 | [-6.4,1.3] |
| N | 1096 |  |  | 1096 |  |  | 1096 |  |  |
|  |  |  |  |  |  |  |  |  |  |
|  | Early neonatal death (per 1000 births) | | | Neonatal death (per 1000 births) | | | Post-neonatal death (per 1000 births) | | |
|  | Coef. | p value | 95% CI | Coef. | p value | 95% CI | Coef. | p value | 95% CI |
| All facility % | 7.5 | 0.05 | [-0.1,15.0] | 8.5 | 0.07 | [-0.8,17.8] | -5.9 | 0.19 | [-14.9,3.0] |
| Small at birth % | 6.3 | 0.14 | [-2.0,14.5] | 10.2 | 0.02 | [1.3,19.2] | 3.4 | 0.58 | [-8.5,15.3] |
| Antenatal care visit median | -0.5 | 0 | [-0.9,-0.2] | -0.7 | 0 | [-1.2,-0.2] | -0.2 | 0.33 | [-0.6,0.2] |
| Multiple birth % | 16.9 | 0 | [12.4,21.5] | 19.6 | 0 | [16.2,22.9] | 4.5 | 0.06 | [-0.1,9.0] |
| Average maternal age | -0.3 | 0.4 | [-0.9,0.4] | -0.3 | 0.44 | [-1.0,0.4] | 0.1 | 0.54 | [-0.3,0.6] |
| Urban % | -2.2 | 0.21 | [-5.7,1.2] | -2.6 | 0.15 | [-6.2,0.9] | 1.2 | 0.3 | [-1.1,3.5] |
| First birth % | -12.8 | 0.05 | [-25.7,0.2] | -14.6 | 0.03 | [-28.0,-1.3] | -9 | 0.33 | [-27.3,9.2] |
| Less than 2 year birth interval % | 16.8 | 0.06 | [-0.7,34.2] | 20.8 | 0.05 | [0.2,41.4] | 20.2 | 0.02 | [3.0,37.4] |
| Mother's primary education % | 1 | 0.81 | [-7.3,9.3] | -0.7 | 0.88 | [-9.4,8.1] | 0.6 | 0.89 | [-7.7,8.9] |
| Mother's secondary education or higher % | -16 | 0 | [-23.9,-8.1] | -19.2 | 0 | [-27.8,-10.5] | -2.4 | 0.08 | [-5.1,0.3] |
| Average annual income | -1.5 | 0.38 | [-5.0,1.9] | -1.2 | 0.51 | [-4.7,2.3] | -2.1 | 0.07 | [-4.5,0.2] |
| South Asia (vs. Sub-Saharan Africa) | 2.9 | 0.45 | [-4.6,10.5] | 4.4 | 0.32 | [-4.3,13.0] | -1.7 | 0.55 | [-7.2,3.8] |
| Middle income country (vs. low income) | 5.8 | 0.03 | [0.5,11.0] | 5.8 | 0.04 | [0.1,11.5] | -3.1 | 0.14 | [-7.1,1.0] |
| N | 1096 |  |  | 1096 |  |  | 1096 |  |  |
