# Supplementary material for: Hospital delivery and neonatal mortality in 37 countries in sub-Saharan Africa and South Asia: An ecological study
Source: PLoS Med. 2021 Dec 1;18(12):e1003843. doi: 10.1371/journal.pmed.1003843 (PMC8635398; doi:10.1371/journal.pmed.1003843)
Supplement: S10 Table — (DOCX) [file pmed.1003843.s011.docx]

**S10 Table**. Interaction model comparing associations in early versus late surveys

|  | Hospital delivery | | | All facility delivery | | |
| --- | --- | --- | --- | --- | --- | --- |
|  | Coef. | p value | 95% CI | Coef. | p value | 95% CI |
| Hospital % among facility deliveries | -15.6 | 0 | [-18.9,-12.3] |  |  |  |
| All facility % | 3.6 | 0.37 | [-4.2,11.3] | 0.8 | 0.89 | [-10.7,12.4] |
| Survey 2015 or before (vs. 2016 or later) | -6.2 | 0.01 | [-10.7,-1.6] | -7.5 | 0.01 | [-12.9,-2.1] |
| Early survey, Hospital % interaction | 13.2 | 0 | [4.9,21.5] |  |  |  |
| Early survey, All facility % interaction |  |  |  | 10.2 | 0 | [3.3,17.1] |
| Small at birth % | 5.5 | 0.14 | [-1.8,12.8] | 7.8 | 0.04 | [0.4,15.2] |
| Antenatal care visit median | -0.2 | 0.28 | [-0.5,0.2] | -0.4 | 0.07 | [-0.8,0.0] |
| Urban % | -0.5 | 0.77 | [-3.7,2.7] | -2.9 | 0.11 | [-6.5,0.7] |
| Multiple birth % | 16.7 | 0 | [12.1,21.3] | 17 | 0 | [12.3,21.7] |
| Average maternal age | -0.1 | 0.71 | [-0.7,0.5] | -0.5 | 0.17 | [-1.2,0.2] |
| First birth % | -4.3 | 0.55 | [-18.5,9.8] | -7.8 | 0.3 | [-22.4,6.9] |
| Less than 2 year birth interval % | 23.5 | 0.01 | [6.1,40.9] | 16.6 | 0.05 | [0.2,32.9] |
| Mother's primary education % | 3.7 | 0.17 | [-1.6,9.0] | 0.1 | 0.98 | [-5.5,5.6] |
| Mother's secondary education or higher % | -12.1 | 0.01 | [-20.7,-3.5] | -15.3 | 0 | [-23.9,-6.7] |
| Average annual income | 0.2 | 0.89 | [-2.2,2.5] | -1.1 | 0.52 | [-4.3,2.2] |
| South Asia (vs. Sub-Saharan Africa) | 4.2 | 0.34 | [-4.3,12.6] | 1.5 | 0.71 | [-6.5,9.6] |
| Middle income country (vs. low income) | 5.8 | 0.06 | [-0.3,12.0] | 5.9 | 0.06 | [-0.3,12.1] |
| N | 1143 |  |  | 1143 |  |  |
