# Supplementary material for: Hospital delivery and neonatal mortality in 37 countries in sub-Saharan Africa and South Asia: An ecological study
Source: PLoS Med. 2021 Dec 1;18(12):e1003843. doi: 10.1371/journal.pmed.1003843 (PMC8635398; doi:10.1371/journal.pmed.1003843)
Supplement: S11 Table — (DOCX) [file pmed.1003843.s012.docx]

**S11 Table.** Random intercept and slopes in sub-Saharan Africa

|  | BLUP random intercept | BLUP random slope | N regions |
| --- | --- | --- | --- |
| Angola | 0.13 | -1.62 | 18 |
| Benin | -0.04 | 0.52 | 12 |
| Burkina Faso | 0.02 | -0.29 | 13 |
| Burundi | -0.10 | 1.25 | 18 |
| Cameroon | 0.33 | -4.15 | 12 |
| Chad | 0.04 | -0.45 | 21 |
| Congo, Dem. Rep. | -0.02 | 0.27 | 11 |
| Congo, Rep. | 0.14 | -1.77 | 12 |
| Côte d'Ivoire | -0.37 | 4.59 | 11 |
| Ethiopia | -0.16 | 1.99 | 11 |
| Gabon | 0.15 | -1.85 | 10 |
| Ghana | -0.07 | 0.92 | 10 |
| Guinea | -0.11 | 1.36 | 8 |
| Kenya | 0.44 | -5.44 | 47 |
| Lesotho | -0.90 | 11.25 | 10 |
| Liberia | 0.10 | -1.23 | 5 |
| Madagascar | 0.13 | -1.58 | 22 |
| Malawi | -0.05 | 0.58 | 28 |
| Mali | 0.11 | -1.42 | 9 |
| Mozambique | -0.16 | 1.99 | 11 |
| Namibia | 0.58 | -7.26 | 13 |
| Niger | 0.17 | -2.15 | 8 |
| Nigeria | -0.19 | 2.35 | 37 |
| Rwanda | 0.16 | -1.97 | 5 |
| Senegal | 0.01 | -0.10 | 14 |
| Sierra Leonne | -0.27 | 3.40 | 4 |
| Swaziland | -0.01 | 0.18 | 4 |
| Tanzania | 0.13 | -1.60 | 30 |
| Togo | 0.05 | -0.56 | 6 |
| Uganda | -0.02 | 0.31 | 15 |
| Zambia | 0.06 | -0.80 | 10 |
| Zimbabwe | -0.26 | 3.27 | 10 |

BLUP: Best linear unbiased prediction

Random slopes and intercepts from a mixed effects model where association of hospital share of facility deliveries on early neonatal mortality is allowed to vary by country
