# Supplementary material for: Hospital delivery and neonatal mortality in 37 countries in sub-Saharan Africa and South Asia: An ecological study
Source: PLoS Med. 2021 Dec 1;18(12):e1003843. doi: 10.1371/journal.pmed.1003843 (PMC8635398; doi:10.1371/journal.pmed.1003843)
Supplement: S12 Table — (DOCX) [file pmed.1003843.s013.docx]

**S12 Table**. Interaction models including contextual variable and covariate interactions

| Country income interactions |  |  |  |  |  |  |
| --- | --- | --- | --- | --- | --- | --- |
|  | Hospital delivery | | | Any facility delivery | | |
|  | Coef. | p value | 95% CI | Coef. | p value | 95% CI |
| Hospital % among facility deliveries | -5.2 | 0.14 | [-12.2,1.8] |  |  |  |
| Middle income country (vs. low income) | -16 | 0.45 | [-57.5,25.5] | 11.2 | 0.59 | [-29.4,51.9] |
| Middle income, Hospital % interaction | -7.8 | 0.05 | [-15.6,-0.1] |  |  |  |
| All facility % | -3.9 | 0.25 | [-10.7,2.8] | 0.6 | 0.90 | [-8.7,9.9] |
| Middle income, All facility % interaction | 10 | 0.03 | [1.1,19.0] | 12.5 | 0.01 | [2.9,22.1] |
| Small at birth % | 3.5 | 0.58 | [-9.0,16.1] | 0.9 | 0.91 | [-14.7,16.4] |
| Middle income, small at birth % | 7.3 | 0.30 | [-6.4,21.1] | 10.2 | 0.22 | [-6.0,26.5] |
| Antenatal care visit median | 0.9 | 0.10 | [-0.2,2.1] | 0.9 | 0.16 | [-0.4,2.1] |
| Middle income, antenatal care visit interaction | -1.2 | 0.05 | [-2.5,0.0] | -1.4 | 0.04 | [-2.8,-0.1] |
| Urban % | -0.4 | 0.93 | [-9.2,8.5] | -2.5 | 0.56 | [-10.8,5.9] |
| Middle income, urban % interaction | -1.8 | 0.69 | [-10.9,7.3] | -0.6 | 0.89 | [-9.5,8.3] |
| Multiple birth % | 26.6 | 0.00 | [20.2,32.9] | 24.2 | 0.00 | [17.8,30.5] |
| Middle income, multiple birth % interaction | -14.6 | 0.00 | [-21.3,-7.8] | -12.8 | 0.00 | [-19.5,-6.1] |
| Average maternal age | -0.8 | 0.08 | [-1.8,0.1] | -0.4 | 0.45 | [-1.3,0.6] |
| Middle income, maternal age interaction | 1.1 | 0.06 | [-0.1,2.3] | 0.7 | 0.18 | [-0.3,1.7] |
| First birth % | 10.7 | 0.67 | [-37.9,59.3] | 22.2 | 0.42 | [-32.2,76.6] |
| Middle income, first birth % interaction | -8.7 | 0.73 | [-57.8,40.5] | -33.1 | 0.23 | [-87.5,21.3] |
| Less than 2 year birth interval % | 6.2 | 0.60 | [-16.7,29.0] | 8.6 | 0.48 | [-15.3,32.5] |
| Middle income, birth interval interaction | 28 | 0.07 | [-2.4,58.4] | 22.1 | 0.13 | [-6.3,50.5] |
| Mother's primary education % | 3.3 | 0.59 | [-8.5,15.0] | -1.3 | 0.83 | [-13.1,10.5] |
| Middle income, primary education interaction | -7 | 0.33 | [-21.2,7.2] | 6.6 | 0.39 | [-8.4,21.6] |
| Mother's secondary education or higher % | -2.1 | 0.67 | [-12.0,7.7] | -9.4 | 0.08 | [-20.1,1.3] |
| Middle income, secondary education interaction | -13.1 | 0.04 | [-25.8,-0.4] | -6.6 | 0.29 | [-18.7,5.5] |
| Average annual income | -0.7 | 0.56 | [-3.0,1.6] | -0.8 | 0.54 | [-3.5,1.8] |
| Middle income, average income interaction | 0 | 0.99 | [-3.9,3.9] | -2.5 | 0.16 | [-6.0,1.0] |
| South Asia (vs. Sub-Saharan Africa) | 3.6 | 0.27 | [-2.9,10.1] | 4.3 | 0.22 | [-2.6,11.3] |
|  |  |  |  |  |  |  |
| Urban interactions |  |  |  |  |  |  |
|  | Hospital delivery | | | Any facility delivery | | |
|  | Coef. | p value | 95% CI | Coef. | p value | 95% CI |
| Hospital % among facility deliveries | -11.9 | 0 | [-16.7,-7.0] |  |  |  |
| Urban regions (vs. rural) | -7.4 | 0.79 | [-62.3,47.5] | -18 | 0.5 | [-70.5,34.6] |
| Urban, Hospital % interaction | -5 | 0.26 | [-13.9,3.8] |  |  |  |
| All facility % | 4.1 | 0.21 | [-2.3,10.5] | 6.8 | 0.07 | [-0.7,14.3] |
| Urban, All facility % interaction | -13.8 | 0.1 | [-30.4,2.9] | -10.4 | 0.21 | [-26.8,5.9] |
| Small at birth % | 1.9 | 0.67 | [-6.7,10.5] | 3.4 | 0.46 | [-5.6,12.4] |
| Urban, small at birth % | 32.5 | 0.02 | [5.3,59.7] | 23.2 | 0.07 | [-2.3,48.7] |
| Antenatal care visit median | -0.5 | 0.02 | [-0.8,-0.1] | -0.6 | 0 | [-1.0,-0.3] |
| Urban, antenatal care visit interaction | 0.6 | 0.05 | [-0.0,1.2] | 0.5 | 0.07 | [-0.0,1.1] |
| Urban % | 3.2 | 0.39 | [-4.0,10.4] | -1.1 | 0.78 | [-8.9,6.8] |
| Multiple birth % | 19.6 | 0 | [15.0,24.2] | 18.4 | 0 | [14.6,22.2] |
| Urban, multiple birth % interaction | -6.9 | 0.31 | [-20.2,6.3] | -5.6 | 0.45 | [-19.9,8.8] |
| Average maternal age | -0.4 | 0.32 | [-1.1,0.4] | -0.4 | 0.23 | [-1.0,0.2] |
| Urban, maternal age interaction | 1.5 | 0.02 | [0.2,2.8] | 1.2 | 0.06 | [-0.0,2.4] |
| First birth % | -0.9 | 0.93 | [-21.4,19.6] | -5.2 | 0.53 | [-21.6,11.2] |
| Urban, first birth % interaction | -20.3 | 0.19 | [-50.8,10.3] | -26.8 | 0.07 | [-56.2,2.6] |
| Less than 2 year birth interval % | 19.1 | 0.12 | [-4.8,43.0] | 18.8 | 0.06 | [-0.9,38.6] |
| Urban, birth interval interaction | -22.1 | 0.14 | [-51.5,7.3] | -23 | 0.16 | [-55.1,9.2] |
| Mother's primary education % | -2.3 | 0.49 | [-8.8,4.2] | 0 | 0.99 | [-6.4,6.5] |
| Urban, primary education interaction | 18 | 0.05 | [-0.3,36.3] | 23.1 | 0.02 | [4.1,42.1] |
| Mother's secondary education or higher % | -12.7 | 0.01 | [-21.9,-3.5] | -15.3 | 0 | [-23.0,-7.6] |
| Urban, secondary education interaction | 11.1 | 0.11 | [-2.6,24.9] | 11.4 | 0.16 | [-4.7,27.6] |
| Average annual income | -0.9 | 0.43 | [-3.2,1.3] | -1.9 | 0.22 | [-4.9,1.1] |
| Urban, average income interaction | -2.6 | 0.36 | [-8.0,2.9] | -0.9 | 0.75 | [-6.2,4.5] |
| Middle income country (vs. low income) | 5.7 | 0 | [2.0,9.4] |  |  |  |
| South Asia (vs. Sub-Saharan Africa) | 3.8 | 0.15 | [-1.4,9.0] | 5.7 | 0.21 | [-3.3,14.8] |
|  |  |  |  |  |  |  |
| Global regions interactions |  |  |  |  |  |  |
|  | Hospital delivery | | | Any facility delivery | | |
|  | Coef. | p value | 95% CI | Coef. | p value | 95% CI |
| Hospital % among facility deliveries | -3.6 | 0.2 | [-9.2,1.9] |  |  |  |
| South Asia (vs. Sub-Saharan Africa) | 30.6 | 0.13 | [-9.1,70.4] | 51.4 | 0.01 | [12.4,90.4] |
| South Asia, Hospital % interaction | -8 | 0.01 | [-14.4,-1.7] |  |  |  |
| All facility % | -0.9 | 0.84 | [-9.9,8.1] | -0.4 | 0.93 | [-9.5,8.6] |
| South Asia, All facility % interaction | 7.6 | 0.18 | [-3.4,18.6] | 11.7 | 0.04 | [0.6,22.8] |
| Small at birth % | 5.8 | 0.51 | [-11.5,23.2] | 5.3 | 0.55 | [-12.0,22.6] |
| South Asia, small at birth % | 3.5 | 0.7 | [-14.1,21.0] | 4.8 | 0.59 | [-12.6,22.1] |
| Antenatal care visit median | 0.2 | 0.36 | [-0.3,0.8] | 0.3 | 0.27 | [-0.2,0.8] |
| South Asia, antenatal care visit interaction | -0.3 | 0.36 | [-0.8,0.3] | -0.6 | 0.04 | [-1.2,-0.0] |
| Urban % | -1.1 | 0.75 | [-7.7,5.5] | -1.7 | 0.59 | [-7.9,4.5] |
| South Asia, urban % interaction | -2.2 | 0.51 | [-8.9,4.4] | -3.1 | 0.32 | [-9.2,3.1] |
| Multiple birth % | 17.5 | 0 | [12.5,22.5] | 17.4 | 0 | [12.5,22.3] |
| South Asia, multiple birth % interaction | -4.3 | 0.13 | [-9.8,1.3] | -4.1 | 0.14 | [-9.4,1.3] |
| Average maternal age | -0.1 | 0.81 | [-1.1,0.9] | -0.1 | 0.82 | [-1.1,0.9] |
| South Asia, maternal age interaction | 0.5 | 0.37 | [-0.6,1.6] | 0.5 | 0.36 | [-0.6,1.6] |
| First birth % | 17.6 | 0.29 | [-14.8,49.9] | 15.1 | 0.36 | [-17.6,47.9] |
| South Asia, first birth % interaction | -20.5 | 0.23 | [-54.3,13.2] | -21.5 | 0.23 | [-56.4,13.4] |
| Less than 2 year birth interval % | 14.4 | 0.22 | [-8.7,37.5] | 14 | 0.23 | [-8.9,36.9] |
| South Asia, birth interval interaction | 20.5 | 0.1 | [-3.7,44.8] | 16.7 | 0.17 | [-7.1,40.4] |
| Mother's primary education % | -0.7 | 0.88 | [-9.1,7.8] | -0.6 | 0.89 | [-9.2,8.0] |
| South Asia, primary education interaction | 18.3 | 0 | [6.9,29.7] | 19.1 | 0 | [8.3,29.8] |
| Mother's secondary education or higher % | -7.7 | 0.14 | [-18.0,2.6] | -8.7 | 0.11 | [-19.4,2.1] |
| South Asia, secondary education interaction | -7.8 | 0.14 | [-18.2,2.5] | -7.7 | 0.16 | [-18.4,3.1] |
| Average annual income | 1.5 | 0.31 | [-1.4,4.4] | 1.5 | 0.33 | [-1.5,4.4] |
| South Asia, average income interaction | -4 | 0.01 | [-6.9,-1.0] | -7 | 0 | [-9.9,-4.1] |
| Middle income country (vs. low income) | 1.9 | 0.37 | [-2.2,6.0] | 1.3 | 0.52 | [-2.7,5.3] |

All models also include year fixed effects
