# Supplementary figures and images for: Hospital delivery and neonatal mortality in 37 countries in sub-Saharan Africa and South Asia: An ecological study
Source: PLoS Med. 2021 Dec 1;18(12):e1003843. doi: 10.1371/journal.pmed.1003843 (PMC8635398; doi:10.1371/journal.pmed.1003843)

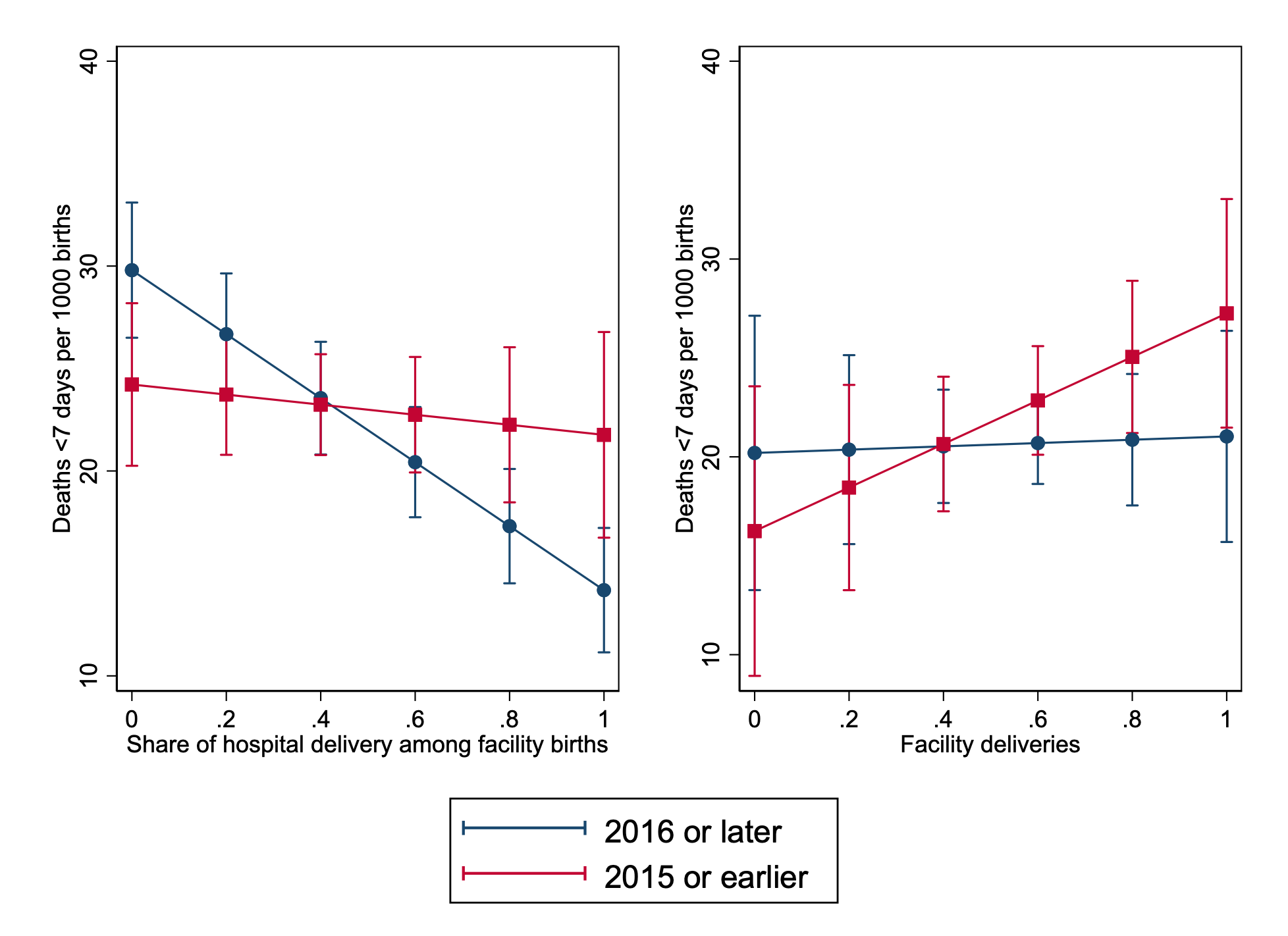

Supplement: S1 Fig — Each graph shows a different multivariable random intercept model that is fully adjusted. Points show the marginal estimate of early neonatal mortality for the given level of hospital deliveries or facility deliveries; bars show 95% confidence intervals. Mortality defined as death within 7 days per thousand births. (TIF) [file pmed.1003843.s014.tif]

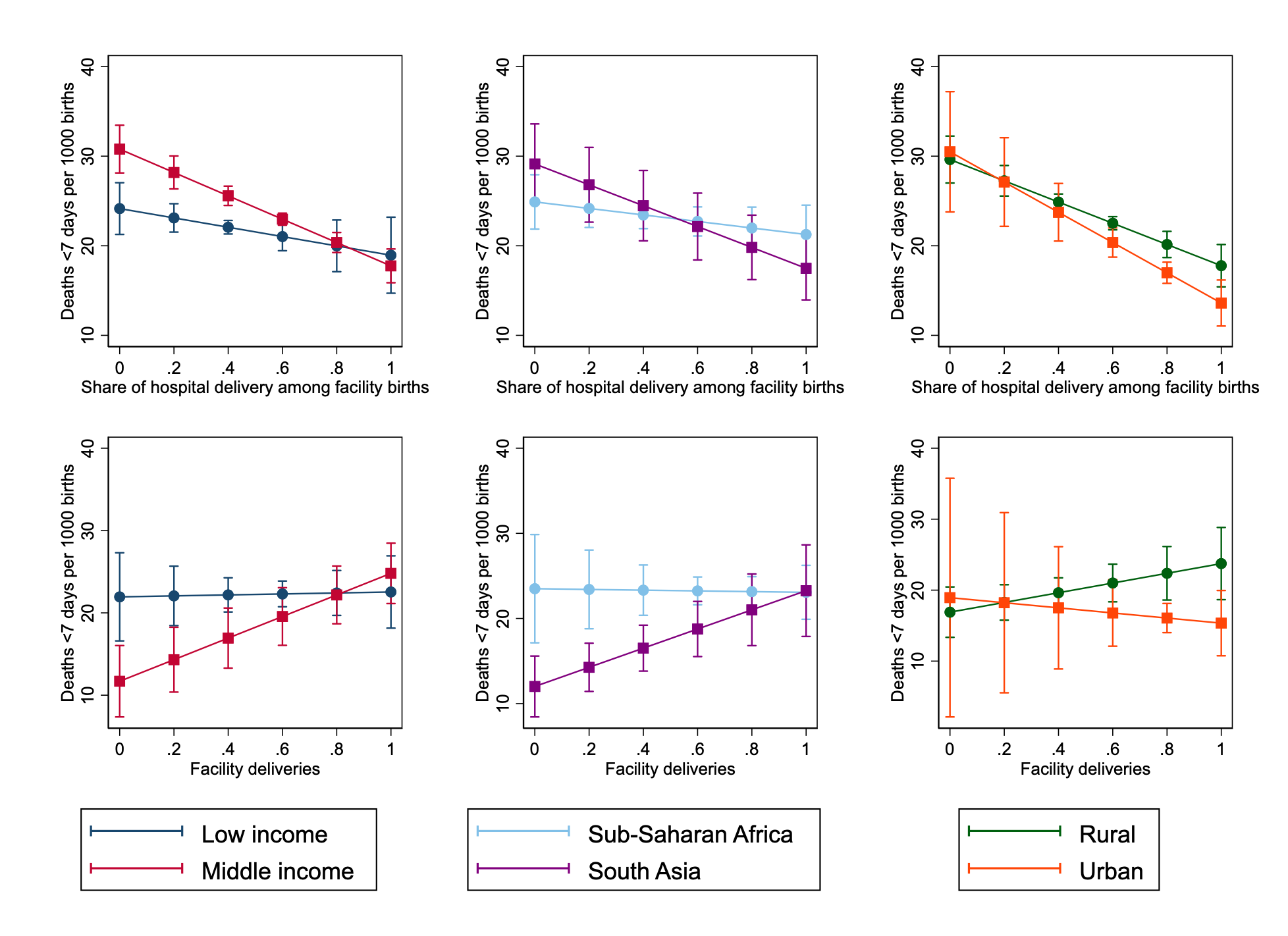

Supplement: S2 Fig — Each graph shows a different multivariable random intercept model that is fully adjusted with all covariates as well as interactions between each of the covariates and contextual variable. Points show the marginal estimate of early neonatal mortality for the given level of hospital deliveries or facility deliveries; bars show 95% confidence intervals. Country income groups defined by the World Bank Group, with low-middle- and upper-middle-income countries combined. Rural regions defined as those with less than 50% of the population classified as urban, urban regions defined as the converse. (TIF) [file pmed.1003843.s015.tif]
