# Supplementary material for: Hospital delivery and neonatal mortality in 37 countries in sub-Saharan Africa and South Asia: An ecological study
Source: PLoS Med. 2021 Dec 1;18(12):e1003843. doi: 10.1371/journal.pmed.1003843 (PMC8635398; doi:10.1371/journal.pmed.1003843)
Supplement: Attachment — Submitted filename: Response to reviewers_v2.docx [file pmed.1003843.s016.docx]

**Requests from the editors:**

1)      Please revise your title according to PLOS Medicine's style. Your title must be nondeclarative and not a question. It should begin with main concept if possible. Please place the study design ("A ecological study,") in the subtitle (i.e., after a colon). For example, “Hospital delivery and neonatal mortality in 37 countries in sub-Saharan Africa and South Asia: An ecological study”

Edited.

2)      Abstract:

a)      Please structure your abstract using the PLOS Medicine headings (Background, Methods and Findings, Conclusions).

Edited.
b)      Please combine the Methods and Findings sections into one section, “Methods and findings”. Please ensure that all numbers presented in the abstract are present and identical to numbers presented in the main manuscript text.

Edited
c)      Please include the actual amounts or percentages of relevant outcomes, not just hazard ratios.

Edited
d)      Please include the important dependent variables that are adjusted for in the analyses.

Edited
e)      Please include p values in addition to 95% CIs.

Edited
f)      In the last sentence of the Abstract Methods and Findings section, please describe the main limitation(s) of the study's methodology.
Edited

3)      Author summary - At this stage, we ask that you reformat your non-technical Author Summary. The Author Summary should immediately follow the Abstract in your revised manuscript. This text is subject to editorial change and should be distinct from the scientific abstract. The summary should be accessible to a wide audience that includes both scientists and non-scientists. Please see our author guidelines for more information: <https://journals.plos.org/plosmedicine/s/revising-your-manuscript#loc-author-summary>.
We have included an author summary following the abstract.

4)      Please conclude the Introduction with a clear description of the study question or hypothesis.
We have rearranged the Introduction to conclude “We aim to answer whether there is an association between regional hospital delivery and regional neonatal mortality. This analysis uses data from 1,143 regions in 37 countries in two high mortality areas, sub-Saharan Africa and South Asia, to examine the ecological relationship between the share of hospital deliveries and neonatal mortality. We further examined how this relationship differs in different contexts.”

5)      Did your study have a prospective protocol or analysis plan? Please state this (either way) early in the Methods section.

No, it does not. We have added this in the Methods section

i)      If a prospective analysis plan (from your funding proposal, IRB or other ethics committee submission, study protocol, or other planning document written before analyzing the data) was used in designing the study, please include the relevant prospectively written document with your revised manuscript as a Supporting Information file to be published alongside your study, and cite it in the Methods section. A legend for this file should be included at the end of your manuscript.

As stated above, we did not develop a prospective analysis plan.

ii)     If no such document exists, please make sure that the Methods section transparently describes when analyses were planned, and when/why any data-driven changes to analyses took place.

We have clarified the timing of decisions throughout the Methods section.

iii)    In either case, changes in the analysis-- including those made in response to peer review comments-- should be identified as such in the Methods section of the paper, with rationale.

6)      Please ensure that the study is reported according to the STROBE guideline, and include the completed STROBE checklist as Supporting Information. Please add the following statement, or similar, to the Methods: "This study is reported as per the Strengthening the Reporting of Observational Studies in Epidemiology (STROBE) guideline (S1 Checklist)." The STROBE guideline can be found here: <http://www.equator-network.org/reporting-guidelines/strobe/>

We have ensured compliance with the STROBE guideline and included it as a supplement.

7)      In your statistical analyses, please use hierarchical/ multilevel models given that nationwide data is likely clustered at various county/ regional levels. The potential clustering of data (e.g., among patients from the same locality or hospital) would result in spurious effect estimates and standard errors. Did you use any approaches to reduce bias due to cluster‐level confounders?

Apologies for the lack of clarity here. All of our main analysis was done at the regional (admin 1) level, which is the lowest level at which DHS levels are designed to be representative. Each region contains on average about 500-1000 households across 25-50 clusters. While the DHS has 5 levels (country – region – cluster- household – individual), we thus use only two (country and region), and account for the clustering of regional outcomes within countries using multilevel modeling.

8)      In statistical methods, please refer to any post-hoc corrections to correct for multiple comparisons during your statistical analyses. If these were not performed, please justify the reasons.

We have included in the methods: “We did not correct for multiple testing in the main analysis, Bonferroni-adjusted p-values are presented in S3 Table.”

9)      Please describe how you selected your adjustment variables.

We have included in the methods: “Covariates included needed to be available from all DHS surveys in our sample, have known associations with neonatal mortality,(Bitew et al., 2020; Houweling et al., 2019) are determined and knowable prior to delivery, and have relevance at the population rather than individual level.”

10)     In the methods section, please provide both 95% CIs and p values in the text.
Edited

11)     Please specify the statistical test used to derive the p values.
Edited

12)     Please include p-values in all tables.
Edited.

13)     Please define the abbreviations in tables and figures e.g. ANC
Edited.

14)     Please indicate in the figure caption the meaning of the bars and whiskers in Figure 1
Edited.

15)     Discussion section, line 13, should be “particular” and not “particularly”

Edited.

16)     Please use the "Vancouver" style for reference formatting and see our website for other reference guidelines <https://journals.plos.org/plosmedicine/s/submission-guidelines#loc-references>.

a)      Please use the PLOS Medicine style reference call outs throughout the text, noting the absence of spaces within the square brackets, e.g., "... child mortality [1,2]."

Edited.
b)      Please ensure that journal name abbreviations consistently match those found in the National Center for Biotechnology Information (NCBI) databases. <https://journals.plos.org/plosmedicine/s/submission-guidelines#loc-references>.

Edited.
c)      Please ensure that all weblinks are current and accessible to date.

Edited.
d)      Please remove “[dataset]” from ref #12

Edited.

17)     Please include line numbers in the next draft
Edited.

**Comments from the reviewers:**

Reviewer #1: "The association between hospital delivery and neonatal mortality: an ecological analysis in 37 countries in sub-Saharan Africa and South Asia" presents an positive association between hospital delivery and reduced neonatal mortality, in particular that a 10% higher share of hospital deliveries was associated with a 6% decrease in early neonatal mortality, or 1.21 per 1000 fewer deaths, from an ecological study design on DHS data from some 680,000 births over 1,143 regions in 37 countries in sub-Saharan Africa and South Asia.

These findings appear not in total agreement with prior studies on much the same DHS source data cited as [5,6], with [6] in particular investigating the same broad regions of sub-Saharan Africa and South Asia also through ecological analyses. These prior work suggest weak/non-significant associations between institutional deliveries/health facilities and neonatal mortality (e.g. from Figure 2B in [6]). The main methodological distinction for this paper appears to be in recognizing different tiers/qualities of health institutions/facilities, in particular between "hospitals" (with capability to perform advanced procedures such as Caesarean deliveries) and "primary-care clinics" (with fewer such capabilities). Increased significance could then be found for neonatal mortality once the comparison is based on (higher-tier) hospital delivery, as suggested from Table S2, rather than on all facilities (which appears correlated with early neonatal births; Table 4). These
associations however appear mixed when analyzed by contextual variables (e.g. Figure 1), and between countries (e.g. Table S4).

Overall, the main conclusions appear plausible, and of importance in motivating better quality of maternal/natal care, especially in less-developed countries. The methodological design also appears appropriate. However, the various analyses might stand to be described in greater detail to facilitate potential replication, and there remain some concerns over the validity of the hospital/clinic definitions used:

Thank you for your careful review and all your suggestions for improvement.

1. In Page 5, it is stated that "The explanatory variable of interest is the share of births occurring in a hospital in each region among those occurring in any facility, whether a hospital, lower-level clinic, or unclassified facility" (i.e. hospital/all facilities); is there a reason why it was not "share of births occuring in a hospital in each region among all births whether in a facility or not" (i.e. hospital/all births)? This might be considered as a secondary analysis.

Thank you for this suggestion. Per this recommendation, we have included a secondary analysis in the supplementary information (S6) that defines the exposure of interest as the proportion of hospital births among all births in the region. We include a line of these findings in the results: “S6 Table shows the exposure defined as share of hospital births among all births: a 10 percentage point increase in overall hospital deliveries is associate with 1.58 fewer early neonatal deaths (p value<0.01; 95% CI 1.23,1.93).”

2. In Page 5, it is stated that "We validated the accuracy of the hospital/clinic categorization by assessing the proportion of Caesarean deliveries in each, as this service is not typically available in lower-level clinics". It might be explained in much greater detail as to how this validation influenced the categorization. For example, was there some threshold of Caesarean deliveries above which the facility would be categorized for purposes of the analyses as a hospital, and otherwise a clinic, and if so, what threshold? And if so, was a single universal threshold applied, since it is later stated that "Caesarean section capacity may in particularly be more common in middle income country hospitals, than in hospitals in low income countries"? Or were facilities not matching their self-definition ignored? In either case, by what extent did any re-categorization differ from the initial categorization, or if no re-categorization was done, by what extent did the original
self-categorization differ from the expected categorization from Caesarean deliveries?

Given that there is no universal definition for hospital and each country has different categorizations for facilities and capabilities for these levels, we primarily relied on the country’s categorizations and defined hospital as any facility that had “hospital” in the DHS category name. However, in some countries, other categories of facilities may have higher level capabilities on par with what may be considered hospital. As Caesarean section is a well reported indicator that suggests presence of these additional capabilities (i.e. availability of an OR, presence of an OB/GYN, blood, etc), we therefore examined the percent of births in each “clinic” facility category that were delivered via Caesarean section, as a high percentage would indicate that the category should actually be classified as a hospital. We used a threshold value of 10%. No facilities were recategorized from hospitals to clinics because of low caesarean rates.

We have included more detail about this validation in the text as requested (lines 187-195) and also included a supplementary table (S1) that shows the percent of caesarean sections delivered by our classification.

3. Related to the above, since the ability to perform Caesarean deliveries (and related advanced services) appears a major factor suggested for the protectiveness of hospital delivery (e.g. "Across five sub-Saharan African countries, for example, quality was poorer in low-volume clinics without Caesarean delivery capacity than hospitals", Page 3), it might be considered to perform brief statistical presentation/analyses relating specifically to Caesarean delivery, if at all possible.

As suggested, we have conducted this analysis, looking at the association between regional c-section rates and neonatal mortality. We have included it below but we have not included it in the study as this relationship has been better studied elsewhere and we intend in this analysis to only use Caesarean section as an indicator of a facility’s capabilities as clarified above.

|  | Early neonatal death (per 1000 births) | | Neonatal death (per 1000 births) | | Post-neonatal death (per 1000 births) | |
| --- | --- | --- | --- | --- | --- | --- |
|  | Coef. | 95% CI | Coef. | 95% CI | Coef. | 95% CI |
| % Delivered by cesarean section | -9.2 | -18.3,-0.1 | -12 | -21.3,-2.7 | 0.1 | -4.6,4.8 |
| All facility % | 8.9 | 1.4,16.5 | 10.5 | 1.3,19.7 | -4.7 | -12.9,3.6 |
| Small at birth % | 6.1 | -0.8,13.1 | 10.2 | 2.8,17.7 | 2.8 | -8.1,13.7 |
| Antenatal care visit median | -0.4 | -0.8,-0.1 | -0.6 | -1.1,-0.1 | -0.3 | -0.6,0.0 |
| Multiple birth % | 16.7 | 12.8,20.6 | 19.1 | 16.3,21.9 | 4.4 | 0.2,8.6 |
| Average maternal age | -0.2 | -0.8,0.4 | -0.2 | -0.8,0.5 | 0.1 | -0.4,0.6 |
| Urban % | -1.4 | -4.9,2.1 | -1.7 | -5.1,1.8 | 1.4 | -0.9,3.7 |
| First birth % | -8.4 | -23.2,6.5 | -9 | -22.5,4.5 | -10.3 | -26.7,6.2 |
| Less than 2 year birth interval % | 17.8 | 0.8,34.7 | 22.6 | 2.8,42.5 | 18.6 | 3.8,33.4 |
| Mother's primary education % | 1.2 | -5.5,7.8 | -0.4 | -7.4,6.6 | 3.6 | -3.8,11.0 |
| Mother's secondary education or higher % | -15.2 | -23.1,-7.3 | -18.3 | -26.9,-9.7 | -1.5 | -4.1,1.2 |
| Average annual income | -1.7 | -5.1,1.7 | -1.3 | -4.8,2.2 | -1.8 | -3.8,0.2 |
| South Asia (vs. Sub-Saharan Africa) | 5.2 | -3.5,13.9 | 7 | -2.9,16.9 | -0.5 | -6.4,5.4 |
| Middle income country (vs. low income) | 2.6 | -2.1,7.4 | 2.4 | -2.7,7.5 | -4 | -7.5,-0.5 |

Models also include survey year fixed effects for years 2009-2018 (Not shown).

For example, while it is stated that "we relied on women's self-report of the level of facility where they delivered. Women may not know the level of the facility or use the term 'hospital' to colloquially mean a health facility" (Page 9), might it be determined as to whether the facilities mentioned did or did not have Caesarean delivery capability, and perform brief analyses according to that firm criteria? Or alternatively perform sensitivity analyses on only facilities for which the status of such capability is known for certain?

From the DHS data, we are unfortunately unable to tell if the facility a woman delivered in was definitively c-section capable if she herself did not report a c-section. We use the c-section thresholds as described above to classify categories of facilities.

4. From Page 5, the set of covariates appears relatively limited, and many reasonable population-level demographic/health confounders apparently not included (e.g. average age of mother at birth, average prevalence of various common diseases/conditions known to affect neonatal mortality, etc.); these might be discussed more comprehensively, if possible.

We have on your suggestion included average maternal age as a covariate in the main model, in addition, we added the percent of first births, which will be a proxy of fertility rates at the regional level. There are very few variables about prevalence of common conditions in the DHS data, however we have included a robustness check in the supplementary material that includes the prevalence of maternal anemia in the subset of countries (28 of 37) that report this variable.

5. It would be highly encouraged to provide more details (e.g. full specification, equations, covariates adjusted for, coefficients found, etc.) about the various analyses and models (two-level random intercept, interaction [also by contextual variables], etc.), as mentioned on Page 6 and Page 7, possibly in the supplementary material. Some details seem already provided (e.g. Table S3 for the interaction model), and might be expanded upon.

We have included clarification in the methods section as requested that these interaction models are adjusted two-level random intercept models and we have provided the full specification in S3.

6. The treatment of stillbirths in these analyses might be explicitly stated, since it appears a significant consideration in prior related studies.

We added to the methods section that stillbirths may be included as early neonatal deaths if the woman reported them as a first day death. We have added to the limitations that stillbirths could not be considered separately as an outcome as stillbirths are recorded inconsistently in versions of the DHS that relied upon a full birth history as opposed to a full pregnancy history and this is regarded as low quality data.

Reviewer #2: Thank you very much for inviting me to review this interesting piece of secondary analysis of demographic health survey in low and middle income countries.
Researchers aim to explore the difference in neonatal mortality (early or late) by the place of birth and provide explanation on what might have been the possible reasons. Researchers have adjusted the possible confounders to assess the association with neonatal mortality and have come to the conclusion that the mortality is high in clinic or primary health care center. From the 32 countries in Sub-saharan Africa and South Asia, using the DHS dataset, 1143 regions were analyzed. The mortality in these 1143 regions were analyzed and also the proportion of hospital birth, primary health care unit and home was analysed. The estimation of the neonatal mortality rate in region was then associated using a linear regression model with the proportion of birth in hospital, primary health care unit and home.
To adjust the confounders, maternal education, average annual income, urban/rural, birth interval, multiple birth, antenatal care visit and sex of baby was done.

This is an interesting way of analyzing the DHS which collects data at individual level using a PPS method and extracting data at sub-national level or regional level to assess mortality with place of birth. I have two major observations and two major feedback to the work
1. The regional mortality estimate does not take into consideration the obstetric complication that is the major factor for early neonatal mortality. As for the late neonatal mortality, there are other postnatal exposures such as infection practices and care of small and sick newborns. I know this is difficult to assess however, DHS does provide information on complication during pregnancy and delivery based on self reporting. Can this been done

Obstetric complication information would be very useful for risk adjustment, and it is available in some specialized DHS surveys such as the one conducted in Ghana in 2017. However, it’s not available in most surveys. This data would also be most helpful to adjust for risk at an individual level, as women who have obstetric complications may be referred to a hospital shortly before or during labor. By conducting the analysis at the regional level, we are including individuals with a range of underlying risks and comparing population level health outcomes.

Postnatal care practices, such as care of small and sick newborns, would take place after the decision of where to deliver has been made, therefore it would not be a confounder between place of delivery and mortality. We have added a sentence to the discussion noting that postnatal care practices particularly for small or sick newborns may be better in hospitals. Our main models also control for the proportion of newborns who were small at birth (a proxy for low birthweight), that may influence whether a woman gives birth in a higher level facility.

2. The regionalized mortality estimate of each region and facility birth takes into a fixed model consideration that all women go to the hospital directly from home, and there is no intra-facility referral in place, which increases the risk of mortality. I know this is not provided in DHS, but a proxy measure is available in DHS questionnaires.

We agree that intra-facility referral data would be very helpful for estimating the association, particularly at an individual level. Our model does not assume that women do not have any intra-facility referral; rather the possibility of this referral is one reason why hospitals have a more challenging case mix and an ecological model is necessary. We are not aware of any proxy measure of intra-facility referral available in the DHS but would welcome it.

3. The high mortality in primary health facilities is postulated, yet not shown in the data might have been due to poor staffing pattern and competency in health facility. Using the three delay care model, the delay in arrival to health facility is one of the major factor for mortality in first referral units. Can the researchers analyse the birth preparedness indicator.

We agree that poor staffing and competency in lower level facilities maybe a major factor contributing to the observed negative association between hospitals and neonatal mortality analysis. While we are not able to examine the quality of care available by level of facility for every country included in the present study, we have included more explicit hypotheses for the relationship in the discussion and cite several papers which do examine birth preparedness in some of the study countries. We also added to the limitations section that we are unable to directly measure quality of care by facility in the study countries: “Third, while we hypothesize that improved quality of care in hospitals may be a key factor for the observed associations, limited cross-country data on quality of care prevents direct measurement.”

4. There has been recent global debates on whether all births should take place in hospitals and primary health care facility should be at outreach clinic, which ANC and PNC by , Roder-DeWan and colleagues in BMJ Global Health. As shown by Ashish KC and colleagues in Journal of global health (Perfect Storm, 2021), that quality of intrapartum care is associated with number of facility birth. I think this has not been considered.

Yes, we agree that there has been lots of discussion on this lately and reference this debate in the discussion section: “Shifting deliveries out of primary care clinics and into facilities that have advanced services to manage complications and sufficient delivery volume to maintain clinical expertise has been proposed (Roder-DeWan et al., 2020).” We added another sentence in the discussion noting the relationship between volume and quality as a proposed hypothesis for why we see the observed associations, “Hospitals may be protective against mortality than other facilities because they have more advanced equipment, infrastructure, and health workers to be able to manage emergencies if they arise; higher volume facilities may also allow greater practice in managing facilities (Kruk et al., 2016; Winter et al., 2017).”

Reviewer #3: This is a critically important topic and although DHS data have limitations in answering the question, this is a well conducted analysis and the adjusted models have controlled for a reasonable set of confounders.

Thank you for this careful review and all your helpful suggestions.

Which countries introduced of voucher schemes or free maternity care, and when? Could this be incorporated in the model? The rationale for this is that introduction of such schemes has commonly increased the number of facility-based deliveries without the corresponding provision of staffing and resources to cope with the increased numbers.

This is an interesting hypothesis and would be a great area for further research. Free maternity care policies have found to be effective in raising facility deliveries in some contexts (i.e. Kenya (Lang’at 2019)) but had more limited effects in others (i.e. Ghana (Amoako Johnson 2015)). Furthermore, “free” maternity care policies have also not always resulted in no costs to clients (Nahar 1998), programs may be implemented sub-nationally, they frequently only affect public sector users, and there are a variety of financing methods to fill the shortfall from client fees. Given the substanatial heterogeneity, it is outside the scope of our study to address. All national policies, including a free maternity policy, are accounted for in the analysis through the country level random effect, but we do not explicitly examine this as a contextual factor.

A '10 percentage point increase in hospital delivery among facility births' is not quite the same thing as suggested by the article title 'The association between hospital delivery and neonatal mortality'. All facility births are compared to home births, for completeness I think the same needs to be presented for hospital births.

Per this recommendation, we have included a secondary analysis defining the exposure of interest as the percent of all births that deliver in hospitals. This is presented in S6 Table and reported in the results: “S6 Table shows the exposure defined as share of hospital births among all births: a 10 percentage point increase in overall hospital deliveries is associate with 1.58 fewer early neonatal deaths (p value<0.01; 95% CI 1.23,1.93).”

We have kept the definition of hospital births as a percentage of facility births as the primary exposure of interest for the main results because we feel this has the most policy salience. Comparing hospital deliveries against a reference of clinic deliveries (rather than clinic and home deliveries) can provide the best evidence of potential gains from redistributing the relative distribution of births among those in facilities. Most countries are already pursuing policies of increasing overall facility births.

In the same light, the sentence in the Discussion 'Across all the categories examined however, share of hospital delivery was more protective than share of facility delivery' really should read 'Across all the categories examined however, share of hospital delivery among facility deliveries was more protective than the overall share of facility delivery'.
Edited.

Please state how much data were missing. Imputing the missingness to be the regional average artificially reduces the width of the distribution and thus potentially increases power of analyses. This is not much of an issue if missingness is rare, but if more than 5 to 10%, could have impacted the findings.

Thank you for this suggestion. We have included a supplementary appendix (S2) that shows the missingness for all variables of interest at the individual level by country. Missingness is very low for deaths (<.01%) and exposure (<6%) in all countries, and under 5% for nearly all covariates. A notable exception is the high level of missingness in Kenya for size at birth (53%); we have consequently included a robustness check that excluded Kenya from the sample in order to account for this (Table S8). We have added a description of this analysis to the methods (lines 238-242)

The analysis is a strong approach, however using DHS data does not allow us to know whether the facility births were those of the small size babies, multiple births etc. Nor were there data on the quality of facilities or quality improvement schemes. Some of these are mentioned in the discussion, but lack of such data is a limitation that should be mentioned. A potential bias is that stillbirths may not be reported as early neonatal mortality at facilities and both stillbirths and early neonatal deaths may also occur at home and not be reported in national statistics or at DHS rounds (<https://pophealthmetrics.biomedcentral.com/articles/10.1186/s12963-020-00225-0>).

We fully agree with these points and have added these two limitations to the discussion section: “Third, while we hypothesize that improved quality of care in hospitals may be a key factor for the observed associations, limited cross-country data on quality of care prevents direct measurement. Finally, this analysis did not consider stillbirths as an outcome because they were inconsistently recorded in versions of the DHS that relied on a full birth history rather than a full pregnancy history (Akuze et al., 2021).”

The argument seems strongest for reducing births at small facilities where resources, staffing and training may be limited - this point is well made in the discussion.

Thank you

Reviewer #4: Thank you for submitting this interesting article on the association between hospital delivery and neonatal mortality.

As you state in your introduction, there have been substantial increases in the proportion of facility-based deliveries, without the corresponding decreases in neonatal mortality.  It's important to try to understand why this is the case.  This article provides some insight into a potential reason for this.

The following are some general comments/suggestions:

Abstract:
You should state in the background section that you are examining the association between place of delivery and neonatal mortality.  Specifically, you seem to be interested in whether delivering a baby in the hospital, clinic or at home is associated with a reduction neonatal mortality. You should also state in the abstract the basis for your analysis and your hypothesis - quality of care in primary care clinics may be sub-optimal.

We have noted in the abstract the basis of our hypothesis: “Widespread increases in facility delivery have not resulted in substantial reductions in neonatal mortality in sub-Saharan Africa and South Asia over the past two decades. This may be due to poor quality care available in widely used primary care clinics.”

What you state in your conclusions is not reflecting your findings.  Indeed, your findings suggests that deliveries at hospital are associated with a reduction in early neonatal mortality rates.  The data you have obtained don't have any information on the quality of care at these hospitals.  Future research should try to understand why tertiary level facilities had lower early neonatal mortality rates.

We have revised the conclusion of the abstract: “Regions with more hospital deliveries as opposed to clinic deliveries have reduced neonatal mortality. Increasing delivery in hospitals while improving quality across the health system should be considered in some areas with high mortality.” We have also included in the limitations section of the paper that we are unable to directly measure the quality of care by level of facility in most study countries due to limited data.

Introduction:
You need to describe what constitutes high quality maternal and newborn care.

We have added the following description to the introduction: “Clinics may be unable to provide high quality maternal and newborn care that can quickly recognize and effectively treat complications when they arise because of limited supplies and equipment, a lack of providers skilled in managing complications, and low volumes which make retaining skills challenging.”

You need to ensure that your objective is accurately stated before your methods  - whether neonatal mortality is associated with place of delivery including primary, lower level clinics and home deliveries.

We have more clearly stated our exposure of interest in the objectives: “This analysis uses data from 1,143 regions in 37 countries in two high mortality areas, sub-Saharan Africa and South Asia, to examine the ecological relationship between the share of facility deliveries occurring in a hospital and neonatal mortality.”

Methods
*       Substantial changes in the proportion of women delivering at health facilities took place during data collection.  You should consider adjusting for this in your analysis, or perhaps stratifying on different time periods.

The year of survey is included as a fixed effect covariate in all our models to account for changing delivery patterns, quality of care, as well as exogenous changes in neonatal mortality. Because the study is conducted at a regional level, we cannot control for the individual year of birth for each of the births included in the sample.

We appreciate the suggestion to include stratification by survey year as another relevant contextual variable and have added this to the supplementary information (S9 Table and S1 Figure), and note the findings in the results section. We find that the associations are stronger in countries that are surveyed in 2016 or after relative to those surveyed 2015 or earlier.

*       The same applies to improving the quality of care between 2009 and 2018, so I feel stratification on time may be warranted.

See above.

*       Some variables you have included as confounders are not correct.  All confounders should be variables that take place before delivery, and not after delivery. If you select variables that include after delivery, they will be on the causal pathway. So the following variables are not appropriate: small at birth, multiple births, male babies.  If the DHS data has information on difficulties in the antenatal period, this may be a relevant confounder.  Also, previous neonatal deaths, stillbirths, miscarriages could be considered.

Although small at birth, multiple birth and male babies are things noted after delivery, they are determined well before delivery and are generally known before delivery; consequently, they may influence the decision of where to deliver. As this is an ecological analysis, we are also adjusting for the prevalence of these covariates in the entire region, thus “small at birth” becomes a proxy for maternal nutrition and risk at the regional level. Upon further consideration, we did decide to drop male births as a covariate as there is little variation at the regional level.

At the regional level, previous neonatal deaths are endogenous with the outcome of interest. Stillbirths and miscarriages are not well reported in the DHS (Akuze et al., 2021), we have added an inability to consider stillbirths as a limitation in the discussion section.

While complications during the antenatal period are not asked in DHS, we have added maternal age and first birth (proxy for fertility rates at the regional level), as two additional variables. We have also included anemia prevalence as a covariate for a subset of countries where it is asked in S7 Table.

*       During the time period that data was collected, women could have more than one delivery.  It is important to account for this in the analysis.  Perhaps some type of GEE model?

As the analysis is at a regional level (the lowest administrative level for which DHS data are representative), all births contribute to the weighted regional mean. Clustering at the mother or cluster level likely lowers the precision of this regional average estimate (i.e, may add a bit of measurement error to the regional mortality estimates), but should not affect our analysis otherwise,

*       You should include the statistical software you used for your analysis as well as the commands you used for your models.  It seems as though you used marginal models.  What command did you use for these models?

We used Stata v15.1 for the analysis. All models were estimated using general least square random effects models with the command xtreg (with xtset at the country). The margins for the interaction models were estimated using the margins command. We have included the statistical software in the methods, per the journal guidelines, but not the specific commands. We have added clarification on the interaction models in the methods and their full specification in Table S3.

*       This is an important paper you should probably reference: <https://www.ncbi.nlm.nih.gov/pmc/articles/PMC5463806/>

Thank you for sharing this interesting study. As it focuses on community-based approaches (home-based counselling and women’s groups) rather than facility-based approaches, its effects are not directly comparable to ours. We have clarified in the discussion of related studies that we are comparing to other studies examining the level or quality of facility of delivery: “The estimated size of the association between hospital delivery and early neonatal delivery is comparable to those seen in other studies examining the level or quality of facility conducting deliveries.”

Minor comments:
Some copy editing is required.
Your discussion includes a recommendation of using implementation science methods to achieve health system redesign.  Could you elaborate further how implementation science could be used to help improve the quality of care at health facilities?

The implementation science methods are proposed as part of future studies to evaluate the effects of health system redesign (i.e. shifting deliveries from clinics to hospitals); they are not meant to improve quality. We have clarified: “Prospective evaluation using implementation science methods can provide needed evidence on how to best achieve successful health system redesign, in which contexts health system redesign may be most beneficial, and how to mitigate for unintended consequences.”

Reviewer #5: The authors have presented a comprehensive analysis of the association between hospital delivery and neonatal mortality in 37 countries in sub-Saharan Africa and South Asia. The manuscript is detailed, well-written, and has the potential to make a valuable contribution to the literature. I have one minor comment about the limitations of the study.  A good number of babies born at health facility dies at the facility without leaving (the facility). Unfortunately, the DHS questionnaires do not single these deaths out. Besides, to be effective in improving neonatal survival in those countries, adding the causes of deaths module to the survey instruments can provided much needed credence to the conclusions of this study.

Thank you for your review. DHS does not distinguish between deaths that occur in or out of the facility; regardless the quality of care provided at the facility during delivery (i.e. early breastfeeding, hand-washing, etc) may still contribute to early deaths outside the facility. It also does not consistently report stillbirths over the surveys examined; we have included this as one of the limitations of our study. We have also included the limitation that we are unable to assess deaths by cause.
