# Supplementary material for: Hospital delivery and neonatal mortality in 37 countries in sub-Saharan Africa and South Asia: An ecological study
Source: PLoS Med. 2021 Dec 1;18(12):e1003843. doi: 10.1371/journal.pmed.1003843 (PMC8635398; doi:10.1371/journal.pmed.1003843)
Supplement: Attachment — Submitted filename: Response to reviewers.docx [file pmed.1003843.s017.docx]

Requests from Editors:

1)      Author summary: Please trim the content under “What did the researchers do and find?” to 2-3 sentences per bullet point.

Each bullet point is from 1-2 sentences.

2)      Please remove the ‘Funding’, “Data availability statement” and “Conflict of interest” from the end of the main text. In the event of publication, this information will be published as metadata based on your responses to the submission form.

Removed.

3)      Please define the abbreviation, ANC, in the table footnotes

Updated.

4)      Please provide the meaning of the bars and whiskers in Figure S1.

Updated.

5)      Please label the Y axis in figure S1 more clearly, i.e., early neonatal mortality (deaths per 1000) or similar\

Updated.

6)      References: Please ensure that journal name abbreviations consistently match those found in the National Center for Biotechnology Information (NCBI) databases

Checked.

Comments from Reviewers:

Reviewer #1: We thank the authors for considering our previous suggestions, and note that the various additional sensitivity analyses generally support the initial claims. A couple of points remain for consideration:

1. The interaction model specification in S4 Table is much appreciated. There is however a concern that the covariates should be further controlled for potential alternative interactions (see: <https://towardsdatascience.com/interaction-analyses-appropriately-adjusting-for-control-variables-d34dfbdd781a>); this might be considered as another robustness check.

Thanks for this suggestion, we have added it to the supplementary material.

2. The formatting of Tables 3 and 4 might however be reconsidered in the latest manuscript, since the final column for post-neonatal death appears to be partially cut off.

Fixed.

Reviewer #2: The researchers have addressed most of the  comments made and highlighted the limitation on examining the quality of care from DHS. However, can the immediate newborn care practice such as skin to skin contact and immediate breast feeding be used as a proxy to quality of care.

We appreciate this suggestion. Unfortunately, skin to skin contact was only added in the newest version of the DHS (version 7), so the variable is only available in 14 out of the 37 study countries. Initiation of immediate breastfeeding is available in all countries, however as a single variable of routine care it is a weak proxy for quality of complications management such as C-section or neonatal resuscitation. We have presented it here for your interest, but we have not included it in the paper.

|  | % Immediate initiation of breastfeeding | | | % Immediate initiation of breastfeeding | | |
| --- | --- | --- | --- | --- | --- | --- |
|  | Coef. | p value | 95% CI | Coef. | p value | 95% CI |
| Hospital % among facility deliveries | 0.00 | 0.48 | [-0.0,0.1] |  |  |  |
| All facility % | -0.10 | 0.11 | [-0.2,0.0] | -0.10 | 0.05 | [-0.2,0.0] |
| Small at birth % | -0.10 | 0.11 | [-0.3,0.0] | -0.10 | 0.11 | [-0.3,0.0] |
| Antenatal care visit median | 0.00 | 0.00 | [0.0,0.0] | 0.00 | 0.00 | [0.0,0.0] |
| Multiple birth % | -0.10 | 0.00 | [-0.2,-0.0] | -0.10 | 0.00 | [-0.2,-0.0] |
| Average maternal age | 0.00 | 0.00 | [0.0,0.0] | 0.00 | 0.00 | [0.0,0.0] |
| Urban % | 0.00 | 0.87 | [-0.1,0.0] | 0.00 | 0.93 | [-0.1,0.0] |
| First birth % | 0.20 | 0.00 | [0.1,0.4] | 0.20 | 0.00 | [0.1,0.4] |
| Less than 2 year birth interval % | -0.40 | 0.00 | [-0.7,-0.2] | -0.40 | 0.00 | [-0.7,-0.1] |
| Mother's primary education % | 0.00 | 0.74 | [-0.3,0.2] | 0.00 | 0.73 | [-0.3,0.2] |
| Mother's secondary education or higher % | 0.30 | 0.00 | [0.1,0.4] | 0.30 | 0.00 | [0.1,0.4] |
| Average annual income | -0.10 | 0.00 | [-0.2,-0.1] | -0.10 | 0.00 | [-0.2,-0.1] |
| South Asia (vs. Sub-Saharan Africa) | -0.10 | 0.11 | [-0.2,0.0] | -0.10 | 0.11 | [-0.2,0.0] |
| Middle income country (vs. low income) | -0.10 | 0.27 | [-0.2,0.0] | -0.10 | 0.31 | [-0.2,0.1] |

Reviewer #3: My comments have been adequately addressed.

Thank you.
